# A novel model to estimate lymph node metastasis in endometrial cancer patients

**DOI:** 10.6061/clinics/2017(01)06

**Published:** 2017-01

**Authors:** Cristina Anton, Alexandre Silva e Silva, Edmund Chada Baracat, Nasuh Utku Dogan, Christhardt Köhler, Jesus Paula Carvalho, Giovanni Mastrantonio di Favero

**Affiliations:** I Hospital das Clínicas da Faculdade de Medicina da Universidade de São Paulo, Instituto do Câncer do Estado de São Paulo – ICESP, Departamento de Ginecologia e Obstetrícia, São Paulo/SP, Brazil; II Asklepios Hospital Hamburg, Department of Advanced Operative and Oncologic Gynecology, GermanyGermany; III Akdeniz University, Department of Obstetrics and Gynecology, Antalya, Turkey

**Keywords:** Endometrial Cancer, Lymph Node Metastasis, Lymphadenectomy, Risk Matrix

## Abstract

**OBJECTIVES:**

To evaluate the postoperative pathological characteristics of hysterectomy specimens, preoperative cancer antigen (CA)-125 levels and imaging modalities in patients with endometrial cancer and to build a risk matrix model to identify and recruit patients for retroperitoneal lymphadenectomy.

**METHODS:**

A total of 405 patients undergoing surgical treatment for endometrial cancer were retrospectively reviewed and analyzed. Clinical (age and body mass index), laboratory (CA-125), radiological (lymph node evaluation), and pathological (tumour size, grade, lymphovascular space invasion, lymph node metastasis, and myometrial invasion) parameters were used to test the ability to predict lymph node metastasis. Four parameters were selected by logistic regression to create a risk matrix for nodal metastasis.

**RESULTS:**

Of the 405 patients, 236 (58.3%) underwent complete pelvic and para-aortic lymphadenectomy, 96 (23.7%) underwent nodal sampling, and 73 (18%) had no surgical lymph node assessment. The parameters predicting nodal involvement obtained through logistic regression were myometrial infiltration >50%, lymphovascular space involvement, pelvic lymph node involvement by imaging, and a CA-125 value >21.5 U/mL. According to our risk matrix, the absence of these four parameters implied a risk of lymph node metastasis of 2.7%, whereas in the presence of all four parameters the risk was 82.3%.

**CONCLUSION:**

Patients without deep myometrial invasion and lymphovascular space involvement on the final pathological examination and with normal CA-125 values and lymph node radiological examinations have a relatively low risk of lymph node involvement. This risk assessment matrix may be able to refer patients with high-risk parameters necessitating lymphadenectomy and to decide the risks and benefits of lymphadenectomy.

## INTRODUCTION

Lymph node (LN) metastasis is the most important prognostic factor in endometrial cancer 1. Approximately 10% of patients diagnosed with clinically apparent early-stage endometrial cancer will be upstaged due to LN involvement 2. Although the role of pelvic lymphadenectomy in early-stage disease was depicted as controversial by two prospective randomized trials, the analysis of the available retrospective data in high-risk patients demonstrated a survival benefit 3,4,5. Currently, the most accurate method to assess the status of the retroperitoneal LNs is surgical removal followed by histopathological analysis. Indeed, this information is crucial for correctly defining the prognosis and tailoring the use of adjuvant oncological therapies. Although the experience of the surgical team with the procedure reduces major intraoperative risks, the risk of postoperative complications, such as deep venous thromboses, potentially fatal pulmonary emboli, chylous ascites formation, lymphocele formation, and lymphedema, remains considerable 6.

Diverse pre- and peri-operative test modalities, such as imaging techniques (magnetic resonance imaging (MRI) and computed tomography (CT)), intraoperative pathological examination of the LNs, sentinel LN biopsy, and final uterine pathological findings, may guide additional (in the same surgery or as a second step surgery) interventions on the LNs 7,8. Moreover, several risk models and scoring systems can be used to avoid unnecessary lymphadenectomies in patients with low-risk disease 8-13.

Nodal dissection requires surgeons with oncological expertise. However, these professionals are frequently not available, particularly in developing countries where access to advanced operative care is very limited. To illustrate, in some parts of Latin America, approximately one-quarter of the population lives in rural areas that are out of reach of oncology centres 14. Therefore, the implementation of standard oncologic care is lacking due to a shortage of qualified surgeons. In this setting, the management of apparently early-stage Endometrial Cancer (EC) could be offered in a two-step approach to avoid unacceptable delays in operative therapy. The rationale behind this oncological concept is the rapid implementation of a simple hysterectomy and bilateral salpingo-oophorectomy (BSO) without upfront nodal dissection as the first-line treatment, followed by subsequent referral of the patients to cancer reference centres to adequately evaluate the necessity of a secondary lymphadenectomy 14,15.

The present study aimed to evaluate the postoperative pathological characteristics of hysterectomy specimens, a preoperative analysis of a tumour marker (cancer antigen (CA)-125) and the imaging results to build a risk matrix model for the identification and recruitment of patients for lymphadenectomy following hysterectomy for seemingly early-stage disease.

## MATERIALS AND METHODS

### Patients and data

The study was conducted in the Instituto do Câncer do Estado de São Paulo do Hospital das Clínicas da Faculdade de Medicina da Universidade de São Paulo, Brazil and was approved by the institutional review board of the hospital (#457.913).

Patients who underwent surgery for epithelial endometrial carcinoma between January 2009 and March 2015 were retrospectively analyzed. Clinical, laboratory, imaging, and histological data were obtained from the electronic medical records. Stromal tumours were excluded from the study.

The following clinical data were recorded: age, race, body mass index (BMI), and the type and histological grade of the cancer in the preoperative biopsy material. The following histopathological parameters were recorded: histological type, histological grade, tumour size, depth of myometrial invasion, presence of lymphovascular space invasion (LVSI), number of removed pelvic and para-aortic LNs, and presence of nodal metastasis. LN involvement was rated as pelvic, para-aortic, or pelvic and para-aortic.

During the preoperative MRI examination, the tumour size, depth of myometrial invasion, and involvement of the pelvic and para-aortic LNs were evaluated. LNs were considered radiologically positive when they were larger than 1.0 cm in the short axis and had a round morphology, MRI signals in T2 sequences similar to those of the tumour, and a restriction to diffusion greater than or equal to the tumour. Only the size and morphological criteria were evaluated during the CT scan.

The CA-125 level was measured at least 24 hours before surgery.

### Statistical analysis

The age, BMI, serum CA-125 level, and tumour size were treated as numeric variables. The race, histological type, histological grade, depth of myometrial invasion, LN involvement by imaging, presence of LVSI, and presence of LN metastasis were treated as categorical variables. A *p*-value <0.05 was considered significant.

The association between the predictive variables and the presence or absence of LN metastasis was assessed by building contingency tables and subsequently evaluating the same variables using the Boschloo test, which is a uniformly more powerful variant of Fisher's exact test 16. The effect size (odds ratio) and its 95% confidence interval were also calculated.

Given the existence of several significant associations between the predictive variables and LN involvement, a stepwise logistic regression was performed to identify the variable combinations with a better predictive performance. The appropriateness of the models was evaluated using the Akaike information criterion 17.

## RESULTS

### Patient population

Among the 408 patients with endometrial cancer who underwent surgery at the institution, three with a diagnosis of carcinosarcoma were excluded from the study. Therefore, 405 patients fulfilled the inclusion criteria and were considered eligible for the analysis. Of these 405 patients, 268 underwent laparoscopy, 132 underwent laparotomy, and 5 underwent robotic surgery. The epidemiological features of the included patients are reported in Table 1.

Most of the analyzed patients (86.7%) were overweight or obese (BMI >25 kg/m^2^), including 90.2% of the patients with type I endometrial cancer (grades 1 and 2 endometrioid carcinoma). Of the patients with type II endometrial cancer (grade 3 endometrioid tumours and tumours with non-endometrioid histology), 83.2% were overweight or obese, whereas 83.1% of the patients with LN metastasis were overweight or obese.

### CA-125

**Figure 1:**
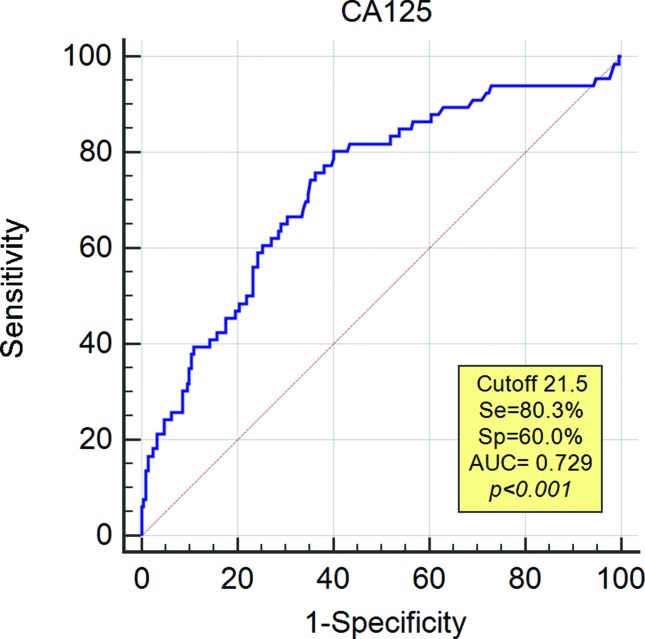
ROC curve for CA-125 in lymph node metastasis prediction. Se: sensitivity, Sp: specificity, AUC: area under the curve.

CA-125 was measured preoperatively in 334 of the 405 patients studied (82.5%). The mean CA-125 value was 21.9 U/mL (range: 4.8–1,577). A receiver operating characteristics (ROC) curve was constructed for the 236 cases in which a systematic lymphadenectomy (pelvic and para-aortic) was performed to determine the best cut-off value to predict LN involvement. The most appropriate cut-off value was 21.5 U/mL, which resulted in 80.3% sensitivity, 60% specificity and 72.9% accuracy (area under the curve (AUC) *p*<0.001) (Figure 1).

### Histological variables

The histological parameters are presented in Table 1. The majority of the patients had low-grade endometrioid histology. More than half of the patients studied had tumours confined to the uterus. LVSI was present in almost one-third of the studied group. The median tumour size was 4 cm.

### Imaging characteristics

Pelvic and abdominal MRI and/or CT images were analyzed to predict pelvic and para-aortic LN metastasis. In total, 385 patients (95.1%) had pelvic images and 377 (93.1%) had para-aortic images available for the analysis.

### Lymph nodes

Of the 405 patients studied, 332 (82%) had at least one LN dissected. The evaluation was complete (pelvic and para-aortic) in 236 (58.3%) patients. Lymphadenectomy could not be performed in 73 (18%) patients due to technical difficulties, poor patient clinical conditions, or complications during surgery. The mean numbers of pelvic and para-aortic LNs were 13 (±10.9) and 6.5 (±8.1), respectively.

Of the 236 patients who underwent complete lymphadenectomy, 74.8% had no LN involvement. Isolated para-aortic LN metastasis was observed in 5 (2.1%) cases. None of the parameters alone or in conjunction were associated with the presence of isolated para-aortic involvement (data not shown).

Myometrial infiltration, lymphovascular invasion, involvement of pelvic LN detected by MRI or CT, and CA-125 levels with a cut-off of 21.5 U/mL were examined by stepwise logistic regression to find combinations of variables better able to predict LN metastasis. These values are shown in Table 2.

A matrix of the probability of LN involvement was created based on the variables selected by logistic regression. A value of (1) was assigned for the presence and (0) for the absence of the variables (Table 3). The risk of LN metastasis ranged from 2.7% in patients with myometrial infiltration <50%, no LVSI, image negative LNs, and a CA-125 level <21.5 U/mL to 82.3% when all four parameters were positive.

### Lymphadenectomy procedure complications

The most relevant complications associated with lymphadenectomy were vascular, neurological, urological and lymphocele complications. Of the patients undergoing lymphadenectomy, 41 (12.3%) had some type of the complications listed above. The patients who did not undergo lymphadenectomy had fewer complications (4, 5.5%), but the difference was not significant.

## DISCUSSION

Retroperitoneal lymphadenectomy is among the most controversial and questionable surgical procedures for the treatment of EC. Nevertheless, this procedure remains an integral part of the surgical therapy for EC according to the latest International Federation of Gynecology and Obstetrics (FIGO) staging system. Certainly, the oncological benefit must be balanced against morbidity, and this equation is difficult to elucidate. Over the last decade, diverse strategies to select patients in whom nodal dissection may be omitted have been proposed and investigated, but none of these strategies have been universally accepted without criticism. Ideally, a risk evaluationmodel should be designed to correctly predict the chances of LN metastasis during the preoperative treatment phase. Following this rational, patients at low risk for lymphatic tumour spread could be more adequately identified, properly informed, and better counselled to reduce the hazards of potentially needless interventions.

In this study, the factors associated with LN involvement in early-stage endometrial cancer were evaluated, and the probability of LN metastasis was assessed using a predictive model. In contrast, with previous studies and other risk matrices, our model incorporates crucial information obtained from the initial hysterectomy. We have worked extensively to create a model of risk assessment by stratifying patients after upfront hysterectomy and salpingo-oophorectomy who did not undergo surgical nodal assessment into risk groups to determine the necessity of a second surgery for retroperitoneal lymphadenectomy. Certainly, definitive pathology of uterine specimen provides unique and reliable data regarding local tumour staging and histological grading compared with preoperative endometrial biopsy and diverse imaging modalities. This information is essential for the construction of more accurate risk matrices for nodal metastasis.

A significant number of patients diagnosed with endometrial cancer live in underserved regions of the world. These women have limited access to adequate on-site oncological surgical care, including a lack of professionals trained to perform retroperitoneal nodal dissection. This situation could lead to an extensive delay in initiating treatment, thereby potentially reducing patient survival. In this population, a primary hysterectomy with BSO regardless of the operative route followed by an evaluation of the pathological uterine parameters may stratify patients more adequately to identify those at high risk for lymphatic spread. These patients should receive a secondary lymphadenectomy in a tertiary oncological referral centre 16,17. After the preoperative imaging study (the status of LNs in pelvic MRI or CT), the uterine pathological findings (deep myometrial invasion < or > 50%), LVSI, and a CA-125 value above 21 U/mL were shown to be associated with nodal metastasis in the logistic regression analysis. Using these parameters, we built an innovative risk assessment matrix to more correctly predict the odds for nodal involvement.

A number of helpful algorithms have been proposed to evaluate the status of LNs 8-13 and to select patients who should receive a retroperitoneal nodal dissection. The most widely used algorithm was proposed by Mariani et al., 2008 18. According to this algorithm, patients with grade 1 or 2 endometrioid carcinoma with superficial (less than half of the uterine thickness) myometrial invasion and a maximal tumour dimension of 2 cm (analyzed by intraoperative frozen section) could be spared from retroperitoneal lymphadenectomy based on their negligible rate of nodal metastasis. When these criteria are applied to women in industrialized nations, approximately one-third can be safely spared nodal dissection 18. However, the application of these criteria to women in developing countries would spare only 8.4% of the patients from undergoing lymphadenectomy. One explanation for this epidemiological phenomenon is that the endometrial cancer diagnosis and therapy more frequently occur in a later phase of the disease in developing countries. Consequently, the vast majority of the available risk assessment algorithms in the literature are of very limited use in developing nations, and a more realistic and adequate model for these populations is certainly needed. Indeed, this issue is one of the strongest points of the present study, in which we tested the utility of several known risk parameters in a different cohort of patients.

Lee et al. proposed a model that incorporated preoperative parameters, including the histological tumour grade, preoperative CA-125 level, local disease extension, and myometrial invasion, to detect patients at low risk for nodal metastasis 12. In this model, the uterine tumour evaluation (lesion size and myometrial and/or cervical infiltration) was assessed by preoperative MRI. The authors also proposed two different cut-off values for CA-125 (28 U/mL for patients aged >50 years and 70 U/mL for patients aged ≤50 years). Use of this model in South Korean women showed that approximately half of the included patients were classified as low-risk, and LN metastasis was not observed in any of the patients after systematic lymphadenectomy. The study concluded that a preoperative prediction system to identify the risk of LN metastasis was feasible and thus models of risk assessment might be useful in preoperative counselling about the costs and benefits of systemic LN dissection. However, this study enrolled a relatively small number of women (110) and was performed in a developed country. In contrast to the study conducted by Lee et al., the tumour size was not directly associated with LN metastasis in our multivariate analysis.

Bendifallah et al. developed an algorithm with the following parameters: age, race, tumour grade, histological subtype, and uterine tumour evaluation, including myometrial invasion and cervical stromal invasion 10. The combined analysis of these five parameters had a very good ability to predict nodal metastasis. This risk assessment model incorporated information from the final uterine histopathologic examination and consequently required surgical treatment in two steps (an initial hysterectomy followed by a possible secondary lymphadenectomy if indicated). This nomogram may be an interesting alternative, particularly for patients at elevated operative risk or with adverse technical surgical conditions. In this scenario, a more accurate model of risk assessment obtained through a primary hysterectomy could provide patients and professionals with a more precise estimation of the probability of LN involvement that might be compared with the risk of a second surgery for nodal dissection. The incorporation of post-hysterectomy histopathological parameters could be a major disadvantage, mainly by increasing morbidity due to the necessity for a reoperation. Nevertheless, we must highlight that up to 25% of the endometrioid tumours preoperatively classified as grade 1 and more than 20% of the carcinomas diagnosed by frozen section as grade 1-2 tumours are upgraded in the final pathological examination 19. Additionally, MRI has a considerable false negative rate for predicting deep myometrial infiltration (up to 13%); its accuracy depends on the equipment quality and professional skill, and the use of this technique increases the cost 9,20. Certainly, this lack of correspondence between the clinical, radiological, and pathological preoperative and postoperative findings leads to an inaccurate risk prediction model.

Pollom et al. evaluated the pathological records and preoperative parameters of 296 EC patients undergoing at least a selective lymphadenectomy 8. In the multivariate analysis, LVSI, deep myometrial involvement, and cervical stromal invasion were significantly associated with LN metastasis. Moreover, the tumour size (>4 cm) was marginally significant. Using these factors, a nomogram was developed in which the absence of all four risk factors implied an irrelevant risk of LN metastasis (<1%). Interestingly, LVSI was strongly associated with nodal involvement, and the authors suggested a preoperative evaluation of this parameter in all cases. However, from a pathological perspective, diagnosing LVSI using limited tumour samples is extremely difficult. Only a full review of the tumour after hysterectomy can provide the precise LVSI status.

The most important limitations of our study are its retrospective nature and the use of data from a single institution. Certainly, the model of risk assessment proposed here requires external validation. Indeed, the matrix generated by our group is grounded in information obtained through an initial hysterectomy and the evaluation of risks concerning a potential second surgery for retroperitoneal lymphadenectomy. In our opinion, the uterine parameters included in our algorithm are more reliable than the information proved by frozen sections. This approach minimizes potential errors and enhances the precision of a risk matrix of LN metastasis in endometrial cancer. The utilization of imaging modalities for uterine staging (especially MRI) requires experienced radiologists for an accurate diagnosis. The interobserver and intraobserver variability was of great importance but could not be assessed in this study.

One strength of the present study is the large number of included patients submitted to systematic retroperitoneal lymphadenectomy. To the best of our knowledge, this study is the first model of risk assessment for endometrial cancer developed and tested in a population from a developing country. Certain social, economic and heath care conditions differ in these areas of the world, and these differences have a substantial oncological impact. The tests and parameters used to construct this risk matrix are commonly available in underserved regions. Moreover, the operative management of the disease in two steps addresses a crucial issue in these nations: the provision of advanced oncological surgical care. This approach offers reliable information for more accurate risk assessment and better selection of patients for nodal dissection. In areas where resources are limited, the implementation of a two-step surgical approach and the use of this matrix will prevent delays in the treatment of endometrial cancer.

In conclusion, patients without deep myometrial invasion and LVSI on the final uterine pathological examination, low Ca-125 values (<21 U/mL) and normal radiological images of the LNs have an extremely low risk of LN metastasis (2.7%) that does not justify the performance of a second surgery for nodal dissection. By utilizing this model of risk assessment, we can more precisely stratify patients at high risk that certainly need retroperitoneal lymphadenectomy. This approach provides crucial and accurate information to enable medical professionals to better counsel patients regarding the necessity for lymphadenectomy, thereby more adequately balancing the risks and benefits, especially in women at elevated surgical risk. Prospective studies utilizing the surgical treatment of type I endometrial cancer in two steps and the external validation of this proposed risk matrix are necessary to verify the results obtained here.

## AUTHOR CONTRIBUTIONS

Anton C conceived the study, collected the data, participated in the analysis, drafted the manuscript and performed the statistical analysis. Silva AS conceived the study and collected the data. Baracat EC, Dogan NU and Kohler C participated in the review of the manuscript. Carvalho JP conceived the study and participated in its design, coordination and drafting. Di Favero GM participated in the drafting and review of the manuscript. All authors read and approved the final manuscript.

## Figures and Tables

**Table 1:** Characteristics of the study population.

Variable	N=405 (100%)
**Age**	
Median (mean)	63 (63.6)
**Range**	31–91
**Race**	
Caucasian	313 (77.3%)
Black	42 (10.4%)
Other	50 (12.3%)
**BMI (kg/m^2^)**	
Median (mean)	27.8 (32.2)
Range	19.2–58
**Histologic type**	
Endometrioid	326 (80.5%)
Serous	60 (14.8%)
Clear cell	19 (4.7%)
**Grade**	
Low	283 (69.8%)
High	122 (30.1%)
**FIGO stage**	
IA	178 (44.0%)
IB	81 (20%)
II	29 (7.2%)
IIIA	22 (5.4%)
IIIB	8 (2.0%)
IIIC1	37 (9.1%)
IIIC2	32 (7.9%)
IVA	2 (0.5%)
IVB	16 (4.0%)
**Myometrial infiltration**	
< 50%	198 (48.9%)
> 50%	196 (48.4%)
Unknown	11 (2.7%)
**Lymphovascular space invasion**	
Yes	128 (31.6%)
No	267 (65.9%)
Unknown	10 (2.5%)
**Tumour size (cm)**	
Median	4
Range	(0–13)

* BMI: Body mass index.

**Table 2:** Logistic regression.

	Lymph node metastasis risk *OR*	CI 95%
**Myometrial infiltration**	2.12	0.86-5.79
**Lymphovascular space invasion**	2.98	1.29-7.41
**Pelvic lymph node involvement by imaging (MRI/CT)**	6.49	2.86-16.9
**CA125 ≥ 21.5 U/mL**	4.01	1.67-11.69

* CI: Confidence interval.

**Table 3:** Risk matrix: probability of lymph node involvement based on the presence or absence of predictive variables.

Myometrial infiltration ≥ 50%	Lymphovascular space invasion	Pelvic lymph node involvement by imaging	CA125 ≥ 21.5U/mL	Probability of lymph node involvement
0	0	0	0	2.7%
1	0	0	0	5.8%
0	1	0	0	7.8%
0	0	0	1	10.1%
1	1	0	0	15.1%
0	0	1	0	15.5%
1	0	0	1	19.3%
0	1	0	1	25.2%
1	0	1	0	27.9%
0	1	1	0	35.3%
1	1	0	1	41.6%
0	0	1	1	42.3%
1	1	1	0	53.6%
1	0	1	1	60.8%
0	1	1	1	68.6%
1	1	1	1	82.3%
